# Chlorfenapyr (A Pyrrole Insecticide) Applied Alone or as a Mixture with Alpha-Cypermethrin for Indoor Residual Spraying against Pyrethroid Resistant *Anopheles gambiae* sl: An Experimental Hut Study in Cove, Benin

**DOI:** 10.1371/journal.pone.0162210

**Published:** 2016-09-02

**Authors:** Corine Ngufor, Jessica Critchley, Josias Fagbohoun, Raphael N’Guessan, Damien Todjinou, Mark Rowland

**Affiliations:** 1 London School of Hygiene and Tropical Medicine (LSHTM), London, United Kingdom; 2 Centre de Recherches Entomologiques de Cotonou (CREC), Cotonou, Benin; University of Crete, GREECE

## Abstract

**Background:**

Indoor spraying of walls and ceilings with residual insecticide remains a primary method of malaria control. Insecticide resistance in malaria vectors is a growing problem. Novel insecticides for indoor residual spraying (IRS) which can improve the control of pyrethroid resistant malaria vectors are urgently needed. Insecticide mixtures have the potential to improve efficacy or even to manage resistance in some situations but this possibility remains underexplored experimentally. Chlorfenapyr is a novel pyrrole insecticide which has shown potential to improve the control of mosquitoes which are resistant to current WHO-approved insecticides.

**Method:**

The efficacy of IRS with chlorfenapyr applied alone or as a mixture with alpha-cypermeththrin (a pyrethroid) was evaluated in experimental huts in Cove, Southern Benin against wild free flying pyrethroid resistant *Anopheles gambiae* sl. Comparison was made with IRS with alpha-cypermethrin alone. Fortnightly 30-minute *in situ* cone bioassays were performed to assess the residual efficacy of the insecticides on the treated hut walls.

**Results:**

Survival rates of wild *An gambiae* from the Cove hut site in WHO resistance bioassays performed during the trial were >90% with permethrin and deltamethrin treated papers. Mortality of free-flying mosquitoes entering the experimental huts was 4% in the control hut. Mortality with alpha-cypermethrin IRS did not differ from the control (5%, P>0.656). The highest mortality was achieved with chlorfenapyr alone (63%). The alpha-cypermethrin + chlorfenapyr mixture killed fewer mosquitoes than chlorfenapyr alone (43% vs. 63%, P<0.001). While the cone bioassays showed a more rapid decline in residual mortality with chlorfenapyr IRS to <30% after only 2 weeks, fortnightly mortality rates of wild free-flying *An gambiae* entering the chlorfenapyr IRS huts were consistently high (50–70%) and prolonged, lasting over 4 months.

**Conclusion:**

IRS with chlorfenapyr shows potential to significantly improve the control of malaria transmission in pyrethroid resistant areas compared to pyrethroid IRS or the mixture. Thirty minute *in situ* cone bioassays are not predictive of the performance of chlorfenapyr IRS under field conditions.

## Background

Indoor residual spraying (IRS) has been one of the major tools contributing to the recent reductions in the burden of malaria [1]. Pyrethroids, owing to their efficacy and low-cost are the most-widely used insecticides in IRS campaigns. Unfortunately, the rapid spread of insecticide resistance in malaria vectors particularly to pyrethroids threatens to undermine control efforts against malaria [2]; many vector populations mainly in West Africa have become very difficult to control with pyrethroids [3]. While some vector control programs have resorted to using organophosphates and carbamates (other WHO-recommended classes) for IRS, the increasing number of reports of resistance to these insecticides in malaria vectors [4–6] remains a major concern. New insecticides with novel modes of action to which there are currently no records of resistance in malaria vectors are thus urgently needed.

Chlorfenapyr, a pyrrole insecticide has shown potential to provide improved control of pyrethroid resistant *An*. *gambiae* [7–9]. It works by targeting the oxidative pathways in the insect’s mitochondria thus disrupting ATP production [9, 10]. Chlorfenapyr shows no cross resistance to existing classes of public health insecticides, hence its novel mode of action makes it a suitable candidate insecticide for targeting malaria vectors that are resistant to current public health insecticides [9].

While such novel insecticides could significantly improve the control of pyrethroid resistant malaria vectors at the moment, any success achieved could be quickly eroded by the subsequent evolution of resistance to these insecticides. To extend their useful life in malaria vector control, resistance management tactics which can delay the evolution of resistance to novel insecticides need to be considered even at the outset of their development. The WHO recommends the use of co-formulated insecticide mixtures for IRS for managing insecticide resistance in malaria vectors [3]. The underlying principle is that insects which are resistant to one component in the mixture can be killed by the companion insecticide thus preventing selection of resistant genotypes and reducing the frequency of phenotypic resistance especially in areas where resistance is still rare [3, 11]. This approach is essentially a population genetics argument. Mixtures of insecticides, like mixtures of antimalarial drugs, can produce additive or even synergistic interactions but these attributes have not been well explored for insecticides under field conditions [3]. Because IRS targets the insect vector at a stage when it is likely to make long contact with the insecticide (fed and resting on the wall), the use of mixtures for IRS could potentially improve vector control efficacy in addition to managing insecticide resistance in malaria vectors [11] and thus need to be investigated.

The present study was designed to assess the efficacy of chlorfenapyr applied alone or as a mixture with alpha-cypermethrin for IRS against pyrethroid resistant *Anopheles gambiae* sl in Cove, Southern Benin following WHO guidelines [12]. A direct comparison was made with alpha-cypermethrin alone. Cone bioassays were performed to assess the residual efficacy of the IRS applications over time.

## Methods

### Study site and experimental huts

The study was performed in a newly constructed experimental hut station in Cove, Southern Benin (7°14′N 2°18′E) belonging to the LSHTM/CREC collaborative Centre. CREC provided authorization to use the experimental hut station. The station is situated at the center of a large rice growing field. The rice paddies provide extensive breeding sites for *An*. *gambiae* throughout the year. The rainy season extends from March to October and the dry season from November to February. The trial was performed between June and September 2015 in experimental huts of the West African design [12]. The experimental huts are built on concrete plinths surrounded by water-filled moats to prevent entry of scavenging ants, and have veranda traps to capture exiting mosquitoes. The walls are made of brick plastered with cement on the inside, with a corrugated iron roof. The huts have a ceiling of palm thatch and four window slits (1-cm gap) on the walls through which mosquitoes enter.

The local vector population in Cove is resistant to pyrethroids and DDT and consists of a mixture of *An*. *colluzi* and *An gambiae* ss, with the latter occurring at lower proportions (23%) and only in the dry season. Molecular analysis revealed a kdr (*L1014F*) allele frequency of 89%. Microarray studies also found CYP6P3 a P450 validated as an efficient metabolizer of pyrethroids to be overexpressed in Cove [13].

### WHO Resistance bioassays

To determine the frequency of resistance to pyrethroids and organochlorines in the wild *An gambiae* Cove vector population during the trial, mosquitoes which emerged from larvae collected from breeding sites at the experimental hut station were tested in WHO cylinder bioassays treated with permethrin 0.75%, deltamethrin 0.05% and DDT 4%. Comparison was made with the laboratory susceptible *An*. *gambiae* Kisumu strain. A total of 100 mosquitoes were exposed in batches of 25 for 1 hour to each insecticide and the control and deaths were scored 24 h later.

### Preparation of experimental huts

Prior to the study, the experimental huts were thoroughly cleaned and the netting on the veranda trap replaced. The windows of the hut were properly sealed with absorbent paper and masking tape to prevent contaminating the mosquito entry points during spraying. Absorbent paper was also placed on the floors of the hut closest to the wall to pick up any run-off during spraying. Spray swaths (with 5-cm overlap) were then marked out on the hut walls using chalk and tape to guide the spray process.

### Application of IRS treatments

The following treatments were tested in the experimental huts:

1. Control (untreated polyester net with 6 deliberate holes)
2. Alpha-cypermethrin IRS applied at 25mg/m^2^
3. Chlorfenpyr (BASF Phantom 21.45%SC) IRS applied at 250mg/m^2^
4. Chlorfenapyr 250mg/m^2^+ Alpha-cypermethrin 25mg/m^2^ mixture IRS

To reduce the impact of hut position on the performance of chlorfenapyr, three different huts were treated with chlorfenapyr IRS alone (250mg/m^2^) and the data collection for the chlorfenapyr IRS treatment was rotated between the huts. Due to the unavailability of huts, it was not possible to do the same for all the treatments.

#### Spraying of experimental huts

The IRS treatments were applied using a Hudson Xpert compression sprayer equipped with a 8002 flat fan nozzle. Following WHO guidelines, an application volume rate of 40ml/ m^2^ was used. The spray man wore full protective clothing and went into the hut with a second person who alerted him of spray timing using a stop watch. Prior to spraying, a guidance pole was attached to the tip of the spray lance to enable the spray man maintain a fixed distance from the hut wall in order to achieve the required swath width during spraying. Each swath was then sprayed from top to bottom using the predetermined lance speed. The palm thatch used on the ceiling of the hut was sprayed lying flat on the floor (outside the hut) and allowed to air dry for 1–2 hours before being fitted to the ceiling of the hut. After spraying each hut, the insecticide solution left in the spray tank was poured into a measuring cylinder to determine the volume sprayed. The actual sprayed volume for the hut treatments did not deviate significantly from the required volume per hut treatment; deviation did not exceed 15% for any of the huts, suggesting that the spraying was accurate.

#### Assessing spray quality

To assess the quality of the spray applications, filter papers (Whatman No 1) measuring 5 x 5cm were taped on each wall in each hut using masking tape prior to spraying the huts. After spraying, the filter papers were placed individually in labelled Aluminium foil and stored at 4°C before analysis at Walloon Agricultural Research Centre (CRA-W), Agriculture and Natural Environment Department, Gembloux, Belgium for chemical analysis using gas chromatography (GC) to assess the application rates.

### Trial procedure and volunteer sleepers

The huts were sprayed on 26^th^ June 2015 and the trial started 2 days later and lasted for 54 nights (8 weeks). Prior to the start of the trial, the verandah trap, hut windows and floors were thoroughly cleaned. Human volunteer sleepers slept in the huts from 9:00pm to 5:00am each night and were rotated through the huts daily using a Latin square design to account for individual attractiveness to mosquitoes. At dawn, the volunteer sleepers collected dead mosquitoes in the room of the hut and all mosquitoes which escaped into the veranda using torches and aspirators. These mosquitoes were then transferred to the laboratory for processing where they were identified according to appropriate identification keys and scored for their blood feeding status, mortality and hut position. Delayed mortality was recorded after 72 hours. Mosquitoes were held at 25±2°C during the observations.

### Residual efficacy of IRS treatments

**a) In situ cone bioassays**

Supplementary cone bioassays were performed to assess the residual effect of the insecticide on the hut walls through the course of the trial. Fifty laboratory maintained susceptible female *An gambiae* Kisumu mosquitoes were exposed every 2 weeks to the treated walls using cone bioassays in batches of 10 mosquitoes per cone. One cone was placed on each wall and one on the ceiling. Mosquitoes were exposed for 30 mins and mortality recorded after 72hours. A laboratory maintained colony of pyrethroid resistant *An gambiae* Cove was also tested for comparison.

**b) Residual mortality of wild mosquitoes**

To assess the capacity of chlorfenapyr to provide prolonged control of wild pyrethroid resistant *An gambiae* in treated huts, data collection in the huts treated with chlorfenapyr IRS alone was continued for 8 more weeks (4 months in total). Data were also collected from the control hut for comparison.

### Outcome measures

The following outcome measures were used to assess the efficacy of the treatments in the experimental huts:

1. Deterrence: percentage reduction in the number of mosquitoes caught in treated hut relative to the number caught in the control hut
2. Exiting rates: due to potential irritant effect of treatments expressed as percentage of the mosquitoes collected from the veranda trap
3. Blood feeding rate: percentage blood fed mosquitoes
4. Inhibition of blood-feeding: reduction in blood-feeding rate relative to the control. This was as follows:
  100(Bfu−Bft)/Bfu
  Where *Bfu* is the proportion of blood-fed mosquitoes in the untreated control huts and *Bft* is the proportion of blood-fed mosquitoes in the huts with a specific insecticide treatment.
5. Mortality rate: percentage of dead mosquitoes after a 72 h holding period.
6. A mass killing effect is desirable to reduce transmission. The overall insecticidal effect of a treatment relative to the number of mosquitoes that would ordinarily enter an untreated control hut was estimated by using the following formula and expressed as a percentage:
  100(Kt−Ku)/Tu
  Where Kt is the number killed in the treated hut, Ku is the number dying in the untreated control hut, and Tu is the total number collected from the control hut.

### Statistical analysis

Proportional outcomes (blood-feeding, exiting and mortality) related to each experimental hut treatment were assessed using binomial generalized linear mixed models (GLMMs) with a logit link function, fitted using the ‘lme4’ package for R (version 2.15.0). A separate model was fitted for each outcome. In addition to the fixed effect of each treatment, each model included random effects to account for the following sources of variation: between the huts; between the sleepers; between the weeks of the trial; and finally an observation-level random effect to account for variation not explained by the other terms in the model (over dispersion).

Differences in deterrence, and mass killing effect between the treatments was analyzed using negative binomial regression based on numbers entering and numbers killed respectively with adjustment for the abovementioned covariates.

### Ethical considerations

The study was approved by the Ethics Review Committee of the London School of Hygiene & Tropical Medicine (approval number 9602) and the Ministry of Health in Benin. Human volunteer sleepers who slept in the huts to attract mosquitoes gave written informed consent prior to their participation and were provided chemoprophylaxis. Through the course of the study, they were examined regularly for signs of fever by a stand-by nurse; any sleepers testing positive for malaria were withdrawn from the study and treated properly.

## Results

### WHO resistance bioassays

The results on the resistance bioassays with the wild Cove vector population of *An gambiae* and the Kisumu strain are presented in Table 1. Mortality of the wild Cove vector population did not exceed 10% with the pyrethroid and DDT treated papers while mortality of the Kisumu laboratory strain was a 100% with all three insecticides.

**Table 1:** Mortality of wild *An gambiae* sl from Cove in WHO resistance bioassays.

Mosquito strain	Insecticide	N exposed	N dead	% Mortality (95% CI)
*An gambiae* Cove (wild resistant)	Control	110	1	1 (0–3)
	Deltmethrin (0.05%)	102	9	9 (4–13)
	Permethrin (0.75%)	104	5	5 (2–9)
	DDT (4%)	98	7	7 (3–11)
*An gambiae* Kisumu (susceptible lab)	Control	101	0	0
	Deltamethrin (0.05%)	99	99	100
	Permethrin (0.75%)	102	102	100
	DDT (4%)	104	104	100

### Hut trial results

**Fig 1:**
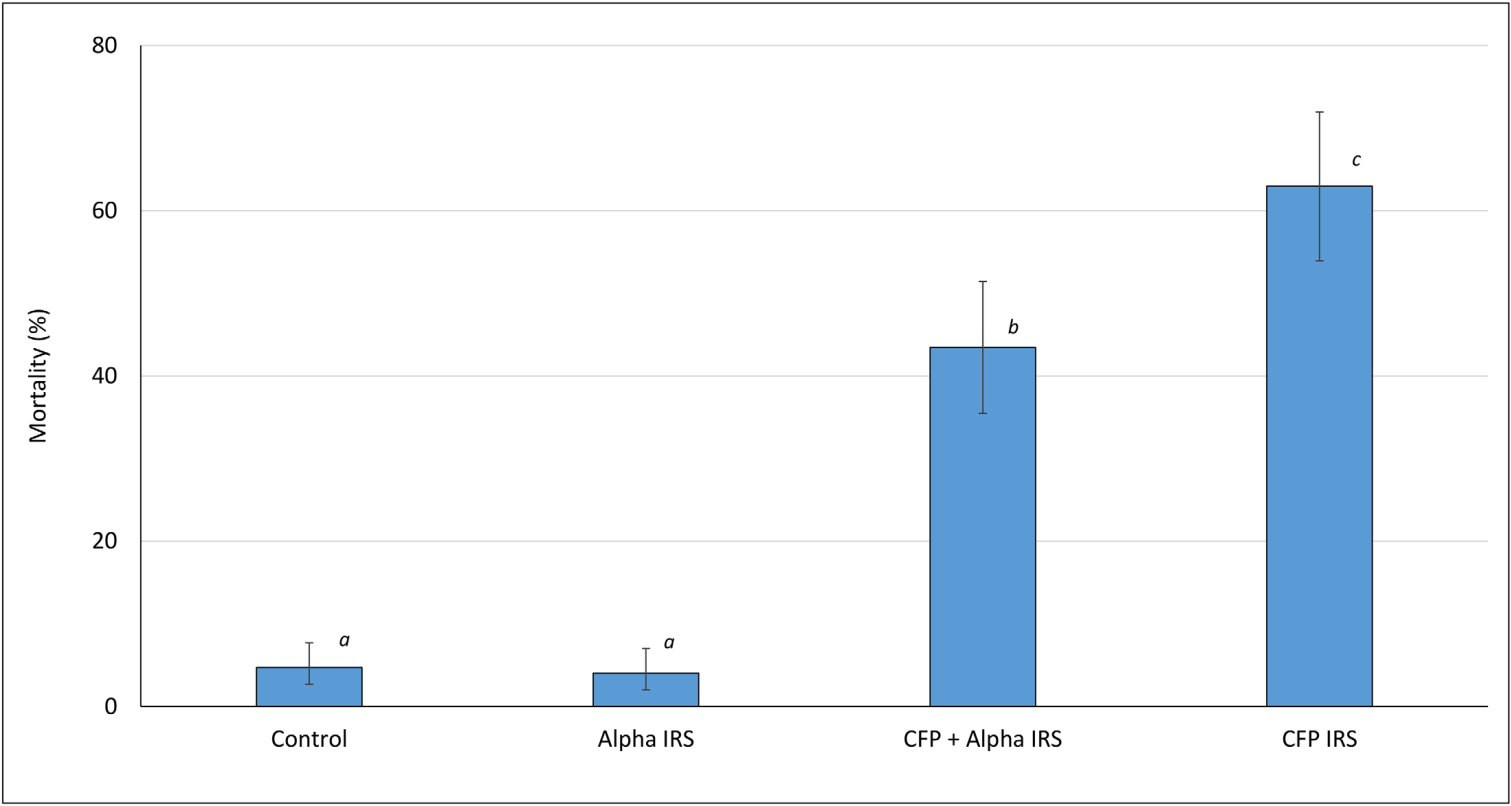
Overall mortality rates of wild pyrethroid resistant *Anopheles gambiae* in IRS-treated experimental huts in Cove, Benin. Bars bearing the same letter label do not differ significantly (P > 0.05). Error bars represent 95% CI. alpha = alpha-cypermethrin, CFP = chlorfenapyr.

The hut trial results are presented in Tables 2–4 and Fig 1. A total of 1382 female *An gambiae* sl were collected in the experimental huts during the trial. The results on hut entry and exiting rates are presented in Table 2. There was no evidence of a hut deterrent effect with alpha-cypermethrin alone or the mixture IRS. Chlorfenapyr alone induced 26% deterrence, however, because the treatments could not be all rotated between the huts, it was not possible to attribute this effect to the treatment with any confidence. Exiting rates were very high across all the treatments; the highest was observed in the hut treated with alpha-cypermethrin IRS probably due to the excito-repellent effect of the pyrethroid.

**Table 2:** Entry and exiting rates of wild pyrethroid resistant *An gambiae* sl in experimental huts in Cove, Benin.

	Control (untreated net)	Alpha IRS	Alpha + CFP IRS	CFP IRS
Total females caught	352	416	355	259
Average caught per night*	6.5^*a*^	7.7^*a*^	6.6^*a*^	4.8^*b*^
% deterrence	-	0	0	26
Total exiting	102	412	307	196
% Exiting*	29^*a*^	99^*b*^	90^*c*^	77^*d*^
95% Conf. Interval	21–38	98–100	85–94	67–85

*Values in the same row sharing a letter superscript do not differ significantly (P > 0.05).

alpha = alpha-cypermethrin, CFP = chlorfenapyr.

**Table 3:** Blood feeding rates of wild pyrethroid resistant *An gambiae* sl in experimental huts in Cove, Benin.

	Control (untreated net)	Alpha IRS	Alpha + CFP IRS	CFP IRS
Total blood fed	237	384	331	209
% Blood fed*	67^*a*^	95^*b*^	96^*b*^	87^*c*^
95% Conf. Interval	54–78	91–97	92–98	77–93
Blood feeding inhibition (%)	-	0	0	0

*Values in the same row sharing a letter superscript do not differ significantly (P > 0.05).

alpha = alpha-cypermethrin, CFP = chlorfenapyr.

**Table 4:** Killing effect of wild pyrethroid resistant *An gambiae* sl in experimental huts in Cove, Benin.

	Control (untreated net)	Alpha IRS	Alpha + CFP IRS	CFP IRS
N dead after 24h	17	18	147	153
% Corrected Mortality*	0	0^*a*^	43^*b*^	63^*c*^
95% Conf. Interval	-	0–1	33–44	52–60
Overall killing effect (%)	-	0	37	39

*Values in the same row sharing a letter superscript do not differ significantly (P > 0.05).

alpha = alpha-cypermethrin, CFP = chlorfenapyr.

There was no significant difference in any of the entomological parameters (proportions exiting, blood feeding and killed) between the 3 experimental huts that were treated with chlorfenapyr IRS alone, hence data reported for this treatment is presented as an average of these 3 huts.

#### Blood feeding rates

Blood feeding rates with the control (untreated net) was 67%. As expected with IRS treatments, blood feeding rates were very high across all the treatments (81–93%), hence there was no evidence of blood feeding inhibition with any of these treatments (Table 3).

#### Mortality rates

Results on mortality are presented in Table 4 and Fig 1. Mortality with the control was 5%. Mortality with alpha-cypermethrin IRS did not differ from the control (4%, P = 0.656). The highest mortality was achieved with chlorfenapyr IRS (63%). Interestingly, mortality rates were significantly lower with the mixture (43%) compared to the chlorfenapyr alone (63%, P<0.001). Alpha-cypermethrin IRS did not induce any killing effect. Overall killing effect was significantly higher in huts treated with the mixture and chlorfenapyr alone (37% and 39% respectively).

### Residual efficacy of IRS treatments

#### In situ cone bioassays

**Fig 2:**
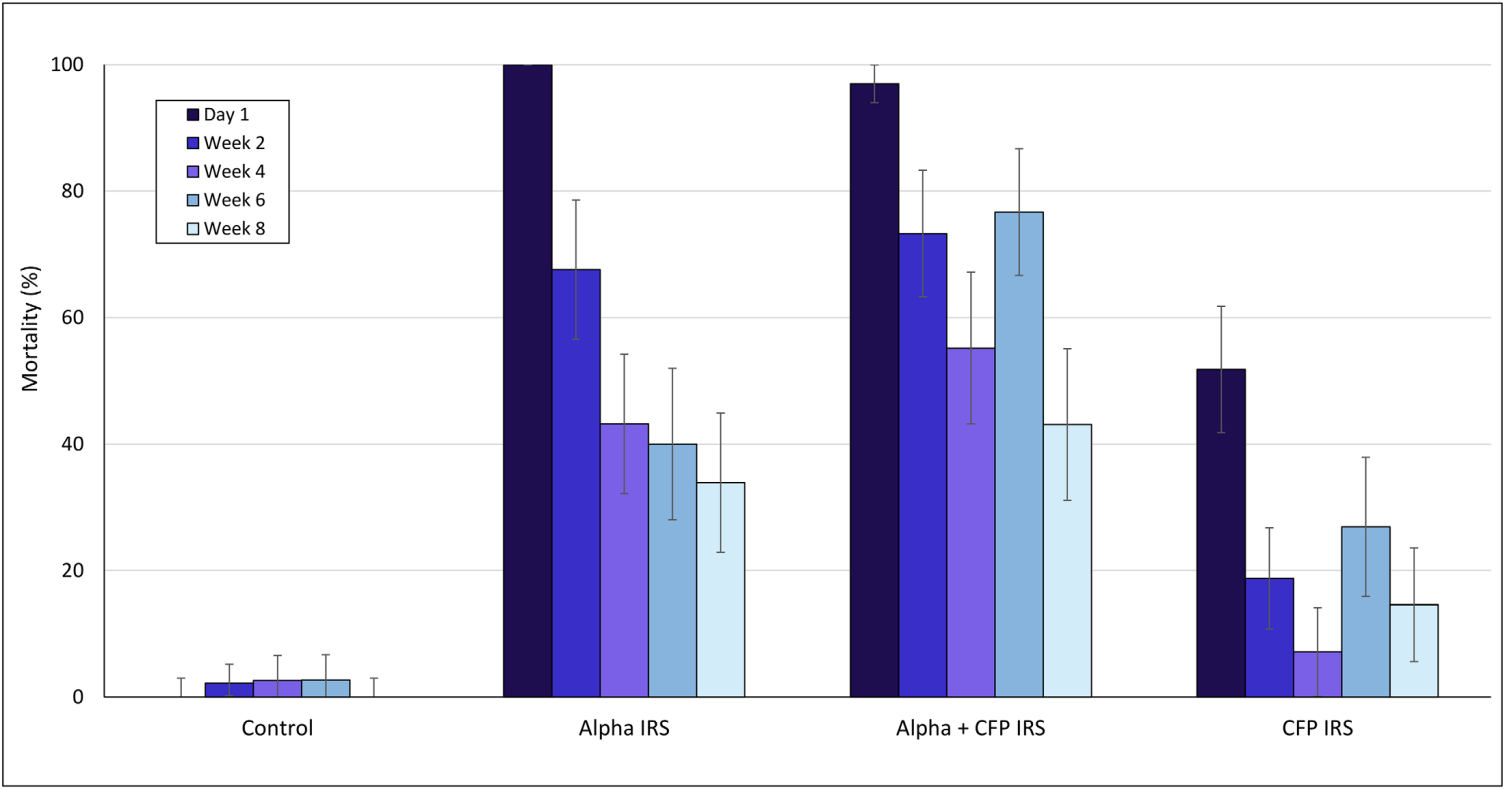
Mortality (%) of susceptible *An gambiae* Kisumu in fortnightly *in situ* cone bioassays in IRS treated experimental huts in Cove. Error bars represent 95% CI. alpha = alpha-cypermethrin, CFP = chlorfenapyr.

**Fig 3:**
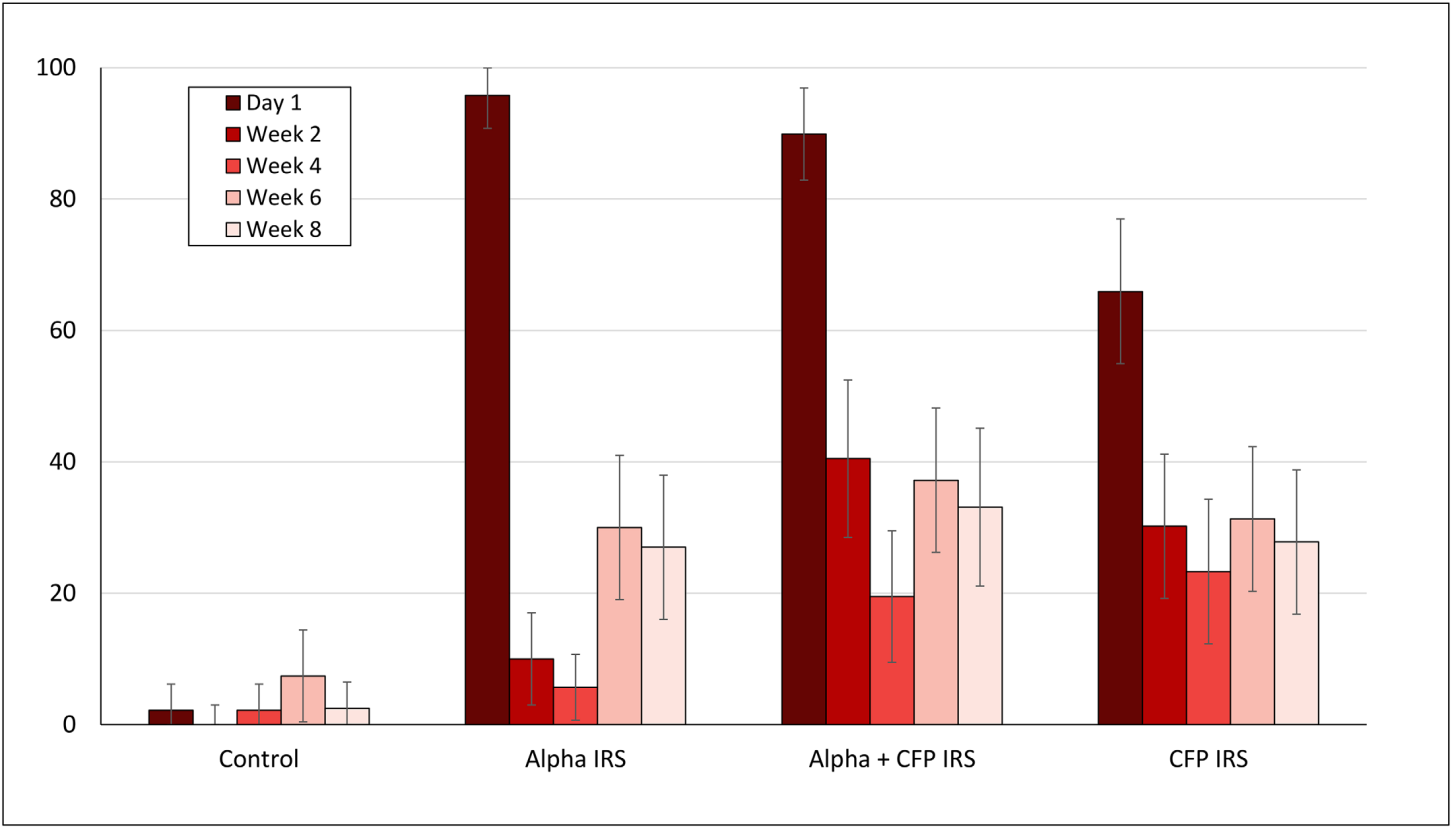
Mortality (%) of pyrethroid resistant *An gambiae* Cove in fortnightly *in situ* cone bioassays in IRS treated experimental huts in Cove. Mosquitoes were collected as larvae from breeding sites close to the experimental huts. Error bars represent 95% CI. alpha = alpha-cypermethrin, CFP = chlorfenapyr.

Mortality rates in *in situ* cone bioassays performed fortnightly on hut wall treatments are presented in Figs 2 and 3 for the susceptible *An*. *gambiae* Kisumu and laboratory-reared pyrethroid resistant *An*. *gambiae* Cove strains, respectively. Overall, the data showed a sudden decline in residual efficacy with both species with all treatments. Mortality rates with the Kisumu strain, were consistently higher with alpha-cypermethrin IRS and the mixture than with chlorfenapyr IRS on day 1 and in subsequent weeks. Cone bioassays with the resistant Cove strain did not also show an improvement in mortality with chlorfenapyr IRS.

#### Prolonged mortality of wild mosquitoes

**Fig 4:**
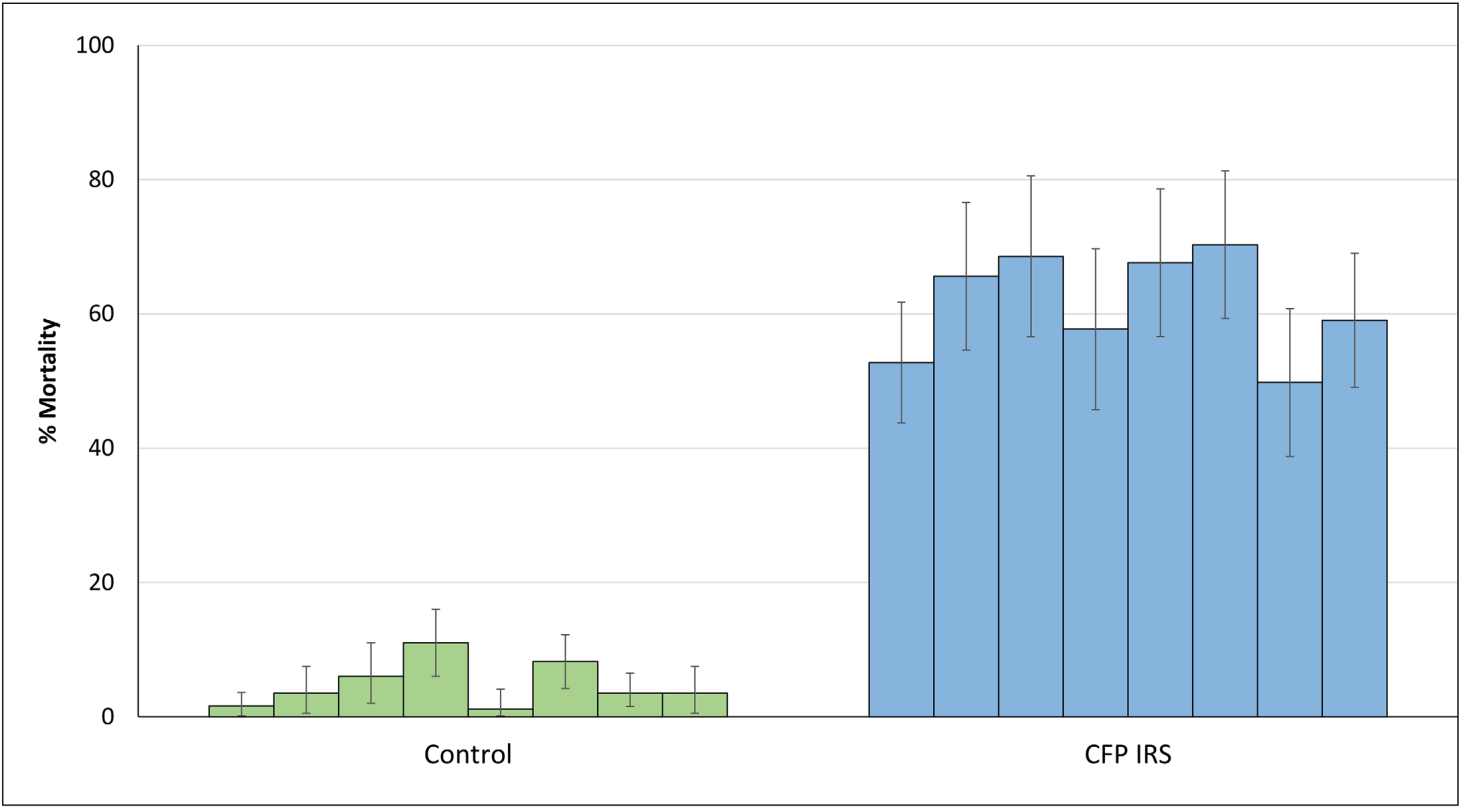
Fortnightly mortality rates of free-flying wild pyrethroid resistant *Anopheles gambiae* entering chlorfenapyr IRS-treated experimental huts in Cove, Benin. Each bar represents mortality data collected over 2 weeks. Data was collected over 4 months. Error bars represent 95% CI. alpha = alpha-cypermethrin, CFP = chlorfenapyr.

The extended mortality rates of wild free-flying *An*. *gambiae* Cove (over 4 months) in experimental huts treated with chlorfenapyr IRS are presented in Fig 4. Fortnightly mortality with chlorfenapyr IRS remained between 50% and 74% throughout this period.

### Assessing spray quality

The results from chemical analysis of filter papers that were placed on the hut walls before spray applications are presented in Table 5. The mean chlorfenapyr content of the filter papers ranged between 227–246 mg/m^2^ for huts sprayed with chlorfenapyr alone and the mixture while the mean alpha-cypermethrin content were 20 mg/m^2^ for the alpha-cypermethrin IRS hut and 21 mg/m^2^ for the mixture. The results were within 20% of the target dose for both insecticides (250 mg/m^2^ for chlorfenapyr and 25 mg/m^2^ for alpha-cypermethrin) thus showing that the IRS treatments were correctly applied.

**Table 5:** Chemical analysis of filter papers from IRS-treated experimental huts in Cove, Benin. Mean insecticide content*.

Hut treatment	Chlorfenapyr content (mg/m^2^)	Relative SD (%)	Alpha content (mg/m^2^)	Relative SD (%)
Alpha IRS	0	-	20	15.9
CFP IRS 1	227	12.6	0	-
CFP IRS 2	210	22.8	0	-
CFP IRS 3	233	15.9	0	-
Alpha + CFP IRS	246	14.5	21	15.5

* Analysis performed by Walloon Agricultural Research Centre (CRA-W), Agriculture and Natural Environment Department, Gembloux, Belgium.

alpha = alpha-cypermethrin, CFP = chlorfenapyr.

## Discussion

The identification of novel IRS insecticides which can effectively control malaria vector populations that have developed resistance to current insecticides especially pyrethroids, is critical for sustaining malaria control. The purpose of this study was to assess the performance of chlorfenapyr, a pyrrole insecticide applied alone or as a mixture with alpha-cypermethrin for IRS against pyrethroid resistant malaria vectors. Results show significantly improved mortality rates with chlorfenapyr and the mixture compared to alpha-cypermethrin alone demonstrating that chlorfenapyr has the potential to prevent malaria transmission in pyrethroid resistant areas better than pyrethroid IRS.

While chlorfenapyr IRS was clearly superior to alpha-cypermethrin IRS against the Cove vector population, the mortality rate achieved was (63%) less than what has been reported with some currently used non-pyrethroid IRS insecticides (>75% mortality) [14, 15]. Similar mortality rates have been achieved with chlorfenapyr IRS at much higher application rates (500mg/m^2^) [7] showing that the maximum impact of the insecticide for IRS will not require doses higher than 250mg/m^2^ as used in the current study. Nevertheless, results on the residual effect of the chlorfenapyr IRS treatments against wild free-flying mosquitoes revealed that these moderate mortality rates were steady and prolonged lasting over 4 months. Prolonged mortality rates with chlorfenapyr IRS lasting >6months have also been observed with wild free-flying pyrethroid-susceptible *An arabiensis* in previous experimental hut studies in Tanzania [16]. This indicates that chlorfenapyr when used for IRS may require less frequent applications to cover transmission seasons compared to some currently available non-pyrethroid IRS insecticides [17]. Because the prolonged effect of chlorfenapyr IRS could only be assessed for 4 months in the present study, further studies investigating its residual activity against pyrethroid-resistant *An gambiae* beyond 4 months will need to be performed. Previous experimental hut studies examining the combination of chlorfenapyr IRS with pyrethroid LLINs against pyrethroid resistant malaria vectors showed significantly improved mortality and blood feeding inhibition with the combined intervention approach compared to chlorfenapyr IRS alone [7]. Chlorfenapyr IRS can therefore be deployed to complement existing pyrethroid LLINs in areas of high pyrethroid resistance.

Intriguingly, the mixture provided significantly lower mortality rates than chlorfenapyr IRS alone. There was no evidence for a negative interaction between the two compounds in bioassay. The higher exiting rates observed in huts with the mixture suggests that the excito-repellent effect of the pyrethroid in the mixture could have reduced the contact time of mosquitoes on hut walls treated with the mixture relative to chlorfenapyr alone, thus preventing them from picking up adequate doses of chlorfenapyr. Previous studies investigating the efficacy of the chlorfenapyr and alphacypermethrin mixture on bed nets demonstrated higher mortality rates (75%) [18] compared to what was achieved with the IRS mixture in the present study (43%). The duration and nature of the contact with the net is different from that of the IRS. Host seeking mosquitoes contact the mixture on a human-occupied bed net while trying to reach the sleeper for a blood meal whereas blood fed mosquitoes alight on a sprayed wall seeking a suitable resting place to digest their meal. Hence the host seeking mosquito may differ in its behavioral response to excito-repellent compounds resulting in longer contact times compared to the IRS. The alpha-cypermethrin and chlorfenapyr IRS mixture used was prepared as a tank mix of both insecticides; the alternative possibility is the more potent chlorfenapyr was masked by less potent compounds. Reformulation of the mixture should address this limitation.

The GPIRM recommends against the use of ad hoc IRS mixtures prepared by simply mixing insecticides. The results of the present study add weight to that view; mixtures for IRS should ideally be co-formulated into a single product using appropriate formulation techniques that ensure similar decline rates for both active ingredients and take into account the differences in their modes of action [19, 20]. Advanced formulations of the chlorfenapyr and alpha-cypermethrin IRS mixture developed by BASF are being evaluated and these could address the limitations of the tank mix.

The present study was designed as a proof of concept to assess the efficacy of chlorfenapyr for IRS and explore its interactions with a contrasting insecticide when applied as a mixture. Given the safety, availability and relatively low cost of pyrethroids, IRS and LLIN mixtures involving pyrethroids are currently the most practical means of adopting the mixture approach for insecticide resistance management. The GPIRM recommendation on use of mixtures for managing resistance is based on the argument that mixtures can delay the selection of resistance provided that the frequency in vector mosquitoes is relatively low in frequency and strength [3]. The conditions in the present study area do not meet this requirement. The frequency and strength of pyrethroid resistance in Cove was too great for a pyrethroid to be effective as IRS, whether on its own or in a mixture with chlorfenapyr. However, insecticide resistance management is a complex process. Demonstrating the resistance management potential of the IRS mixture will require more carefully designed community randomized studies that assess the impact of the IRS mixture on resistance gene frequency over extended periods or years.

Despite the steady and prolonged fortnightly mortality rates of wild free-flying pyrethroid resistant *An gambiae* entering the experimental huts with chlorfenapyr IRS (50–70% for up to 4 months), the 30-minute *in situ* cone bioassays showed a rapid decline in mortality of the Cove strain to <32% after only 2 weeks. Hence 30-min cone bioassays conducted in daytime were not predictive of the residual efficacy of chlorfenapyr IRS against wild free-flying mosquitoes entering the experimental huts at night. Chlorfenapyr is a non-neurotoxic slow-acting insecticide which acts on the metabolic pathway of mosquitoes by disrupting respiratory pathways in mitochondria (through oxidative phosphorylation) after activation by cytochrome P450s [21]. Mosquito circadian rhythm and environmental temperature both play a large role in determining the metabolic status of the insect. Recent developmental studies on chlorfenapyr ITNs showed improved mortality at night (when mosquitoes are intrinsically more active) compared to tests performed during the day [22]. Oxidation of chlorfenapyr to the toxic metabolite and its effect on mitochondria and proton transfer could explain the improved and steady mortality rates achieved in the experimental huts where mosquitoes fly freely and contact the chlorfenapyr IRS walls *ad libitum* at night unlike the residual 30-min daytime cone bioassays. The current study therefore raises further questions over the suitability of the 30 min cone bioassay in the evaluation of non-neurotoxic IRS insecticides and highlights the need for current WHOPES testing guidelines to be tailored to the characteristics and mode of action of each insecticide. Identifying novel public health insecticides for IRS poses a major challenge to chemical companies, hence it is important that our evaluation systems are relevant. Studies are on-going to identify alternative laboratory IRS evaluation systems for slow acting non-neurotoxic insecticides like chlorfenapyr which take into account the circardian rhythm and other metabolic aspects of the mosquito that would affect efficacy.

## Conclusion

IRS with chlorfenapyr shows potential to significantly improve and prolong the control of malaria transmission in pyrethroid resistant areas compared to pyrethroid IRS. Greater levels of transmission control can be achieved with IRS with chlorfenapyr alone compared to mixtures of insecticides that include pyrethroid in situations where resistance to pyrethroid is high in frequency and strength. The 30-minute cone bioassay was deficient as a tool for assessing the residual efficacy of chlorfenapyr IRS. Chlorfenapyr IRS can be applied on its own to control pyrethroid resistant malaria vectors or deployed to complement pyrethroid LLINs in pyrethroid resistant areas.

## Supporting Information

S1 Table Experimental hut data, excel file. (XLSX)
